# 25-Hydroxyvitamin D is closely related with the function of the pancreatic islet β cells

**DOI:** 10.12669/pjms.293.2982

**Published:** 2013

**Authors:** Jingjing Guo, Zhengda Xiao, Xia Xue, Xin Liu, Yong Lu, Xiao Yin, Kun Ma

**Affiliations:** 1 Jingjing Guo, School of Medicine, Department of Endocrinology, Jinan Central Hospital Affiliated to Shandong University, Jinan, 250013, P R China.; 2 Zhengda Xiao, Department of Endocrinology, Jinan Central Hospital Affiliated to Shandong University, Jinan, 250013, P R China.; 3 Xia Xue, Department of Endocrinology, Jinan Central Hospital Affiliated to Shandong University, Jinan, 250013, P R China.; 4 Xin Liu, Beijing University of Chinese Medicine, Beijing, 100029, P R China.; 5 Yong Lu, Department of Endocrinology, Jinan Central Hospital Affiliated to Shandong University, Jinan, 250013, P R China.; 6 Xiao Yin, Department of Endocrinology, Jinan Central Hospital Affiliated to Shandong University, Jinan, 250013, P R China.; 7 Kun Ma, School of Medicine, Shandong University, Jinan, 250012, P R China.

**Keywords:** Vitamin D, Diabetes mellius, Type 2, Pancreatic islet β cell

## Abstract

***Objectives:*** This study is to investigate the relationship between 25-Hydroxyvitamin D (25-OH-D) and pancreatic islet β cell function under different glucose tolerance statuses in China.

***Methodology:*** Totally, 180 patients with type 2 diabetes mellitus (DM group), 178 patients with impaired fasting glucose/impaired glucose tolerance (IFG/IGT group), and 160 normal control subjects (NGT group) were included to measure their body parameters and biochemical parameters. In oral glucose tolerance test, fasting serum 25-OH-D was assessed by the enzyme-linked immunosorbent assay (ELISA). Homeostasis model assessment for insulin resistance (Homa-IR), insulin acuity index (IAI), β-cell function index (Homa-BCF) as well as secretion index (IS) were determined.

***Results:*** The levels of 25-OH-D, IAI and Homa-BCF in the DM group and IFG/IGT group were significantly lower than that in NGT group (P < 0.05). Homa-IR in DM group and IFG/IGT group was significantly higher than that in the NGT group (P < 0.05). Pearson correlation analysis and partial correlation analysis showed that 25-OH-D was positively correlated with fasting insulin (FINS) and Homa-BCF (P < 0.05). Multiple stepwise regression analysis showed that 25-OH-D was one of the influential factors of pancreatic islet β cell function in patients with type 2 diabetes mellitus.

***Conclusions:*** Our results suggest that 25-OH-D is closely related with the function of the pancreatic islet β cells and is one of the influential factors of pancreatic islet β cell function.

## INTRODUCTION

Type 2 diabetes is a multigenic hereditary disease that results from the interaction of genetic factors and environmental factors. It has brought heavy social and economic burden and is a serious worldwide public problem that threatens people's health. Therefore, studying the pathogenesis of diabetes is important for the prevention of this disease.

Nowadays the relationship between vitamin D levels and the risk of diabetes has become a hotspot of research, although most of these researches mainly **focus** on Type 1 (insulin-dependent) diabetes. Banmgard et al.^1^ found a marked decrease in the levels of 1,25-dihydroxy-vitamin D_3_ (1, 25-(OH)_2_D_3_) at onset of Type 1 diabetes compared to normal controls. Ziegler et al.^2^ reported that administration of 1, 25-(OH)_2_D_3_ in newly diagnosed patients with type 1 diabetes can protect the function of pancreatic islet β cell effectively.

Moreover, in both animal models and human, deficiency of vitamin D has been confirmed to affect the synthesis and secretion of insulin. By studying animal models, Norman et al.^3^ found that vitamin D deficiency inhibited the insulin secretion of the rat pancreatic islets. Pancreases from vitamin D-deficient rats which were supplied with glucose and arginine showed reduction in insulin secretion compared with those supplied with vitamin D. Therefore, Vitamin D supplementation can restore the ability of insulin synthesis. Santos et al.^4^ found that vitamin D supplementation can increase insulin secretion and decrease blood glucose concentration in obese Wister rats (classic type 2 diabetes animal model). In human studies, Palomer et al.^5^ reported that vitamin D deficiency might predispose to glucose intolerance. Vitamin D supplement improved insulin secretion in type 2 diabetes with established hypovitaminosis D. The expression of vitamin D receptors (VDR) and vitamin D-binding proteins (DBP) in pancreatic tissue could be regulated by the levels of vitamin D, and promote the ability of insulin synthesis and secretion. In 2005, Zhou et al.^6^ concluded that vitamin D supplementation had a protective effect on patients with latent autoimmune diabetes (LADA) who had been administrated for oral hypoglycemic agents or insulin. However, there are few reports on the relationship between the levels of active vitamin D and the function of pancreatic islet β cell in patients with type 2 diabetes mellitus. This study is to investigate the relationship between 25-Hydroxyvitamin D (25-OH-D) and pancreatic islet β cell function under different glucose tolerance statuses in China.

## METHODOLOGY

***Patients: ***The outpatients and inpatients were collected from the Department of Endocrinology, Jinan Central Hospital affiliated to Shandong University between 1 January, 2008 and 1 May, 2012. Prior written and informed consent was obtained from every patient and the study was approved by the ethics review board of Shandong University. The subjects were divided into three groups. The DM group included 180 patients (98 males and 82 females) with type 2 diabetes mellitus, the IFG/IGT group included 178 subjects (90 males and 88 females) with impaired fasting glucose/imparied glucose tolerande, and the NGT group included 160 normal control subjects (78 males and 82 females). Diagnosis of patients was performed according to the World Health Organization (WHO) 1999 diagnostic criteria for diabetes. Patients with those disease such as acute or chronic infectious disease, stress state, hepatic and renal disease, coronary disease were eliminated. Moreover, patients who were treated with vitamin D and calcium within one year, and patients who once received insulin treatment were excluded. All of the subjects were free of osteoporosis as detected by dual X-ray absorptiometry. 

***Measurements: ***Human body parameters, including height, weight, waist circumference, hip circumference and blood pressure of the subjects were measured by the same researcher under the same measurement tools. The body mass index (BMI) and waist-hip ratio (WHR) were calculated.

Biochemical parameters were measured as follows. The venous blood were extracted from research subjects that had been fasting 12 h before the test (forbidden to drink 8 h before the test), and the total cholesterol (TC), triglyceride (TG), low density lipoprotein cholesterol (LDL-C), high density lipoprotein cholesterol (HDL-C), fasting blood glucose (FBG) were detected by automatic biochemistry analyzer (Hitachi, Japan). Fasting insulin (FINS) in the plasma was detected by radioimmanoassay (RIA). Blood glucose was detected by glucose oxidase method. Fasting serum 25-OH-D was assayed by the enzyme-linked immunosorbent assay (ELISA) on BIO-RAD Model 680 microplate reader (USA).

Oral glucose tolerance tests (OGTT) were performed as follows. Subjects after fasting were supine and rest for 30 minutes, and 75 g glucose was orally given. Venous blood glucose and insulin were measured at 0 h, 0.5 h, 1 h, 2 h and 3 h. Insulin resistance, insulin acuity, β-cell function and the secretion were assessed according to the homeostasis model assessment. 

The indexes used for calculation were as follows.

BMI (kg/m^2^) = weight/height^2^

Insulin resistance index (Homa-IR)** =** (FINS×FPG)/22.5

Insulin acuity index (IAI)** =** Ln^1/(FINS×FPG)^

β-cell function index (Homa-BCF )= 20×FINS/FPG-3.5

Secretion index (IS)** =**FINS/FPG

***Statistical analysis: ***Data were analyzed by SPSS software 17.0 (SPSS, Chicago, IL), and presented as mean ± SD. The values were in abnormal distribution and are statistically analyzed after logarithmic transformation. Variance and covariance analyses were performed for comparison between groups. Pearson correlation analysis and partial correlation analysis were performed to study the correlation between 25-OH-D and other parameters, and the multiple stepwise regression analysis was performed for identification of the influence factors for islet function. P < 0.05 was considered to be statistically significant.

## RESULTS

To investigate the difference in general clinical data and biochemical indexes under different glucose tolerance statuses, measurements were performed among subjects from the DM group with type 2 diabetes mellitus, the IFG/IGT group with impaired fasting glucose/impaired glucose tolerance, and the NGT control group, and statistical analyses were performed. As shown in Table-I, there was no statistical difference in age, BMI, WHR, blood pressure and FINS among the three groups (P > 0.05). However, there were some differences among the three groups. TG and LDL-C in DM group were significantly higher than those in the NGT group (P < 0.05). TC and Homa-IR in both DM group and IFG/IGT group were higher as compared with those in the NGT group (P < 0.05). The levels of 25-OH-D and IAI in the DM group and the IFG/IGT group were significantly lower than that in the NGT group (P < 0.05). IS in the DM group was significantly lower than in the NGT group (P <0.01). FBG and Homa-BCF among the three groups had significant difference (variance analysis, P < 0.05).

**Table-I:** General clinical parameters and biochemical indicators in 3 groups (mean ± SD).

*Characteristic*	*NGT*	*IFG/IGT*	*DM*
N (M/F)	180 (98/82) ^c^	178 (90/88) ^c^	160 (78/82) ^c^
Age (years)	52.80±4.86	53.43±7.45	53.05±1.46
BMI (kg/m^2^)	23.84±1.31	24.72±1.40	24.79±1.46
WHR	0.85±0.04	0.87±0.24	0.88±0.03
SBP (mmHg)	121±11	122±10	123±12
DBP (mmHg)	77±8	78±6	80±5
TC (mmol/L)	5.14±1.13	5.61±1.02^a^	5.65±1.03^a^
TG (mmol/L)	1.88±1.21	1.93±1.36	2.47±1.65^ a^
LDL-C (mmol/L)	3.13±0.92	3.34±1.66	3.45±0.97^ a^
Serum FPG (mmol/L)	5.38±0.42	6.38±0.57^ a^	7.98±3.18^ab^
FINS (μU/ml)	12.90±5.75	16.12±8.80	14.26±10.61
Homa-IR^*^	1.07±0.62	1.38±0.54^ a^	1.39±0.26^ a^
IAI^*^	-4.15±0.53	-4.48±0.54^ a^	-4.48±0.89^ a^
Homa-BCF^*^	4.75±0.54	4.58±0.545^ a^	4.29±0.95^ ab^
IS^*^	0.82±0.56	0.79±0.54	0.49±0.59^ ab^
Serum 25-OH-D (nmol/L)	59.84±27.65	39.08±8.67^ a^	33.88±12.64^ a^

***Note:*** NGT, Normal glucose tolerance group; IFG/IGT, Impaired fasting glucose/impaired glucose tolerance; DM, Type 2 diabetes mellitus group; M, Male; F, Female; BMI, Body mass index; WHR, Waist-hip ratio; SBP, Systolic blood pressure; DBP, Diastolic blood pressure; TC, Total cholesterol; TG, Triglycerides; LDL-C, Low density lipoprotien-cholesterol; FPG, Fasting plasma glucose; FINS, Fasting insulin; Homa-IR, Homeostasis model assessment for insulin resistance; Homa-IR, Homeostasis model assessment for insulin resistance; IAI, insulin acuity index; Homa-BCF, β-cell function index; IS, Secretion index; ^*^, the values were in abnomal distribution and are statistically analyzed after logarithmic transformation; ^a^, P < 0.05 compared with the NGT group;^ b^, P < 0.05 compared with the IFG/IGT group; ^c^, the number of subjects in each group.

As shown in Table-II, 25-OH-D was negatively correlated with BMI, FBG, FINS and Homa-IR, whereas positively correlated with IAI. After adjusting BMI, WHR, TC, TG, LDL-C and Homa-IR, 25-OH-D was positively correlated with FINS, Homa-BCF and IS. Next we performed multiple stepwise regression analysis by setting Homa-BCF and IS as the dependent variables respectively, while 25-OH-D, BMI, TC, TG, LDL-C, Homa-IR as independent variables. The results showed that 25-OH-D was one of the influential factors of pancreatic islet β cell function in patients with type 2 diabetes mellitus. (B_1 _= 0.012, SE_1_=0.006, β_1_=0.275, *P*_1_<0.05; B_2_=0.016, SE_2_=0.004, β_2_=0.228, *P*_2_<0.05).

**Table-II:** Pearson correlation analysis (r value) and partial correlation analysis (r’ value) of the relationship between 25-OH-D and various indicators

	r	r’
BMI	-0.418 (P=0.002)	-----
TC	-0.032 (P=0.763)	-----
TG	-0.120 (P=0.4256)	-----
LDL-C	-0.037 (P=0.787)	-----
FPG	-0.202 (P=0.010)	-0.181 (P=0.124)
FINS	-0.247 (P=0.023)	0.287 (P=0.012)
Homa-IR	-0.305 (P=0.005)	-----
IAI	0.319 (P=0.003)	-0.004 (P=0.957)
Homa-BCF	0.126 (P=0.417)	0.260 (P=0.039)
IS	-0.084 (P=0.425)	0.288 (P=0.024)

***Note:*** BMI, Body mass index; TC, Total cholesterol; TG, Triglycerides; LDL-C, Low density lipoprotien-cholesterol; FPG, Fasting plasma glucose; FINS, Fasting insulin; Homa-IR, Homeostasis model assessment for insulin resistance; Homa-IR, Homeostasis model assessment for insulin resistance; IAI, insulin acuity index; Homa-BCF, β-cell function index; IS, Secretion index; r’, relationship after adjusting BMI, WHR, TC, TG, LDL-C and Homa-IR.

## DISCUSSION

Deficiency of vitamin D has been confirmed to affect the synthesis and secretion of insulin. However, the role of 25-Hydroxyvitamin D in patients with type 2 diabetes mellitus through regulating the function of pancreatic islet β cells is not fully understood. Our results suggested that 25-OH-D is related to the function of pancreatic islet β cells in patients with type 2 diabetes mellitus. In human, vitamin D receptor (VDR) gene was located on 12q13 and had multiple restriction enzyme digestion sites. Through an experiment of 49 healthy Caucasian subjects underwent oral glucose tolerance test (OGTT), Chiu et al^7^ found that the Fok I polymorphism at the VDR gene locus is associated with insulin sensitivity. Li Hui-Min et al^8^ reported that the prevalence of VDR gene genotype frequencies and allele frequencies in patients with type 2 diabetes mellitus was significantly different from that in control subjects. The allele f and genotype ff were more common in patients with type 2 diabetes mellitus than in nondiabetic subjects, with the relative risk (RR) of 3.9 (P < 0.05). Therefore, polymorphism of VDR gene is associated with type 2 diabetes mellitus in Han Chinese, and allele f may be a susceptible gene contributing to the development of type 2 diabetes mellitus in Han Chinese.

We also investigated the function of pancreatic β-cells in type 2 diabetes patients by OGTT and found that 25-OH-D was negatively correlated with FBG and positively correlated with IAI. After adjusting BMI, WHR, TC, TG, LDL-C and Homa-IR, 25-OH-D was positively correlated with FINS, Homa-BCF and IS. Multiple stepwise regression analysis showed that 25-OH-D was one of the influential factors of pancreatic islet β cell function in patients with type 2 diabetes mellitus. Thus we concluded that the increase of blood glucose levels significantly down regulated the levels of serum 25-OH-D, which indicated that vitamin D deficiency might lead to decreased glucose tolerance and pancreatic β-cell dysfunction. The mechanism of this may be that the activated vitamin D activates the L-type calcium channels on β cells to promote the release of insulin and insulin receptor substrate tyrosine phosphorylation, initiating insulin signal transduction. On the contrary, deficiency of vitamin D leads to the close of calcium ion channels, or the disruption of phosphorylation of insulin receptor substrate, which represses the insulin signal transduction and **reduces** the synthesis and secretion of insulin directly. Moreover, some studies have showed that the process of pancreatic β-cell apoptosis in patients with type 2 diabetes was a result of cell factor mediated immune inflammation response in recent years.^9^

Insulin resistance and insulin secretion defects were two risk factors in type 2 diabetes. Our study found that Homa-IR in DM group and IFG/IGT group were higher as compared with that of NGT group (P < 0.05) and serum 25-OH-D was negatively correlated with Homa-IR ,which indicated that Vitamin D deficiency increased the individual risk of insulin resistance and T_2_DM. Pittas et al.^10^ found that HOMA-IR increased along with the levels of vitamin D and calcium, which further supports the hypothesis that calcium and vitamin D affect the risk of type 2 diabetes by modifying insulin resistance. In insulin-sensitive tissues, calcium is necessary for insulin-mediated intracellular reaction. In the major target tissues of insulin, changes in the concentration of calcium may impair the insulin signaling and the function of glucose transporter-4, which leads to peripheral insulin resistance.^11^ Lee et al.^12^ also confirmed that 1, 25-(OH)_2_D_3_ decreased the formation of fat and reduced insulin resistance in peripheral tissues through directly suppressing the expression of peroxisome proliferator-activated receptor γ protein and inhibiting adipocyte differentiation of 3T3-L1 preadipocytes.

In summary, vitamin D may play an important role in the development of type 2 diabetes. Vitamin D may influence β cell function through multiple pathways, thus affecting the synthesis and secretion of insulin, as well as the sensitivity to insulin. Vitamin D deficiency may cause and worsen insulin resistance, and aggravate the damage to β cell function. The mechanism of how 25-OH-D influence blood glucose and pancreatic β-cell function is not yet fully understood. Further studies are still needed to clarify the role of vitamin D in patients with type 2 diabetes in the molecular level.

## Authors Contribution

Jingjing Guo: Conceived, Designed and did statistical analysis & editing of manuscript

Xia Xue, Xin Liu, Yong Lu, Xiao Yin & Kun Ma: Did data collection and manuscript writing.

Zhengda Xiao: Did review and final approval of manuscript.
